# Modulation of plant architecture by the miR156f–OsSPL7–OsGH3.8 pathway in rice

**DOI:** 10.1093/jxb/ery273

**Published:** 2018-07-25

**Authors:** Zhengyan Dai, Jiang Wang, Xiaofang Yang, Huan Lu, Xuexia Miao, Zhenying Shi

**Affiliations:** 1 Key Laboratory of Insect Developmental and Evolutionary Biology, Institute of Plant Physiology and Ecology, Shanghai Institutes for Biological Sciences, Chinese Academy of Sciences, Shanghai, China; 2 National Key Laboratory of Plant Molecular Genetics, Institute of Plant Physiology and Ecology, Shanghai Institutes for Biological Sciences, the Chinese Academy of Sciences, Shanghai, China; 3 University of the Chinese Academy of Sciences

**Keywords:** Auxin, miR156f, *Oryza sativa*, OsGH3.8, OsSPL7, plant architecture, tiller

## Abstract

Tiller number and plant height are two of the main features of plant architecture that directly influence rice yield. Auxin and miR156, an extensively studied small RNA (smRNA), are both broadly involved in plant development and physiology, suggesting a possible relationship between the two. In this study, we identified a rice T-DNA insertion cluster and dwarf (*cd*) mutant that has an increased tiller number and reduced plant height. The T-DNA insertion was in close proximity to the *miR156f* gene and was associated with its up-regulation. Plants overexpressing *miR156f* resembled the *cd* mutant. In contrast, plants overexpressing an miR156f target mimic (MIM156fOE) had a reduced tiller number and increased height. Genetic analysis showed that *OsSPL7* is a target of miR156f that regulates plant architecture. Plants overexpressing *OsSPL7* had a reduced tiller number, while *OsSPL7* RNAi plants had an increased tiller number and a reduced height. We also found that OsSPL7 binds directly to the *OsGH3.8* promoter to regulate its transcription. Overexpression of *OsGH3.8* and *OsGH3.8* RNAi partially complemented the MIM156fOE and *cd* mutant phenotypes, respectively. Our combined data show that the miR156f–OsSPL7–OsGH3.8 pathway regulates tiller number and plant height in rice, and this pathway may allow crosstalk between miR156 and auxin.

## Teaser

miR156f modulates rice plant architecture by direct binding to the *OsGH3.8* promoter through its target OsSPL7, to allow crosstalk with the auxin signaling pathway.

## Introduction

Rice (*Oryza sativa*) is a staple food for nearly half of the world’s population. To address the imbalance between increasing population demands and decreasing availability of arable land, many breeding programs focus on improving grain yield and plant architecture to increase productivity. Tiller number and plant height are two important agronomic traits that directly contribute to plant architecture (Zhang, 2007).

Auxin was the first plant hormone to be identified, and is involved in an extraordinarily broad spectrum of developmental and physiological processes (Sauer *et al.*, 2013). Examples of these include axillary bud dormancy, ectopic shoot development, and leaf angle (Yoshida *et al.*, 2012; Zhao *et al.*, 2013). Because of the universal role of auxin and its rapid action, auxin levels are tightly controlled by the concerted co-operation of biosynthesis, metabolism, and transport in plants. In Arabidopsis, nearly 95% of auxins exist as amino acid and protein conjugates (Ljung *et al.*, 2002; Woodward and Bartel, 2005). Some of these molecules act as intermediates for degradation, and some provide a reusable metabolite pool for free indole-3-acetic acid (IAA) (LeClere *et al.*, 2002; Rampey *et al.*, 2004). Auxin rapidly and transiently induces the accumulation of at least three families of transcripts: *SMALL AUXIN-UP RNAs* (*SAURs*), *GH3*-related transcripts, and *AUXIN/IAA* (*Aux/IAA*) family members (Woodward and Bartel, 2005). The *GH3* family of acyl-acid-amido synthetases catalyzes the ATP-dependent formation of amino acid conjugates with plant hormones, including auxins and jasmonates (Chen *et al.*, 2010). This family is present in various plant species, and includes 19 members in Arabidopsis and 13 in rice (Staswick *et al.*, 2002; Terol *et al.*, 2006; Jain and Khurana, 2009). GH3 enzymes are grouped into three families according to their sequence homology and acyl-acid substrate preference. Two of these families are present in rice: class I and II, which mediate jasmonate and salicylic acid conjugation, respectively (Staswick *et al.*, 2002; Westfall *et al.*, 2010).

Small RNAs (smRNAs) are non-coding RNAs of ~22 nucleotides (nt) in length. There are two broad categories of smRNA, miRNAs and siRNAs (Aravin *et al.*, 2003; Bartel and Bartel, 2003; Bartel, 2004). We have witnessed an explosion in our understanding of smRNAs across different species. Improved understanding of the mechanisms by which smRNAs interact with their target RNAs and the important roles of smRNA in various developmental and physiological processes have revealed that smRNAs are simple in their core mechanism but have profound functions (Nelson *et al.*, 2003; Bartel, 2009). Moreover, the sequences, mechanisms, and functions of miRNAs are conserved across species (Jones-Rhoades, 2012).

Plant miRNAs target key developmental regulators, many of which are transcription factors (Rhoades *et al.*, 2002; Jones-Rhoades *et al.*, 2006). Among them, miR156 has a wide scope (Wang and Wang, 2015) that includes transition from vegetative to reproductive growth and flowering time (Wu *et al.*, 2009; Huijser and Schmid, 2011; Wang, 2014; S. Yu *et al.*, 2015), embryo patterning (Nodine and Bartel, 2010), gibberellin-mediated flowering (Yu *et al.*, 2012), age-dependent flowering (Bergonzi *et al.*, 2013), gynoecium differential patterning (Xing *et al.*, 2013), leaf development (Xie *et al.*, 2012), trichrome distribution (Xue *et al.*, 2014), lateral root development (N. Yu *et al.*, 2015), plant architecture (Bhogale *et al.*, 2014; Chen *et al.*, 2015), biosynthesis of secondary metabolites such as anthocyanin and sesquiterpene (Gou *et al.*, 2011; Z.X. Yu *et al.*, 2015), ovary and fruit development (Ferreira e Silva *et al.*, 2014), and developmental timing (Cho *et al.*, 2012). In rice, ectopic expression of miR156b and miR156h results in dwarfism, a small panicle, and delayed flowering (Xie *et al.*, 2006). Together with miR529 and miR172, miR156 regulates vegetative and reproductive branching (Wang *et al.*, 2015). One target of miR156, *SQUAMOSA PROMOTER BINDING PROTEIN-like14* (*SPL14*), regulates optimal plant architecture and has great potential for improving rice yield (Jiao *et al.*, 2010; Miura *et al.*, 2010; Springer, 2010). Another target, *OsSPL16*, regulates rice seed size and quality (Wang *et al.*, 2012). Furthermore, miR156 responds to various abiotic and nutritional stresses (Jia *et al.*, 2010; Zhou *et al.*, 2010; Nischal *et al.*, 2012; Wang *et al.*, 2013; Cui *et al.*, 2014; Stief *et al.*, 2014; Song *et al.*, 2015).

The functional activity and universality of both miR156 and auxin led to the hypothesis that there is crosstalk between them in regulating plant architecture. In this study, we identified a cluster and dwarf (*cd*) mutant that had underlying up-regulation of miR156f. Genetic analysis of miR156f and the *OsSPL7* gene showed that the miR156f/*OsSPL7* module regulates plant architecture, with ectopic expression of miR156f and down-regulation of *OsSPL7* leading to plants with an increased number of tillers and reduced height. Moreover, ectopic expression of an miR156f target mimic (MIM156f) and up-regulation of *OsSPL7* dramatically reduced the number of tillers. Further biochemical and genetic analyses confirmed that OsSPL7 directly regulates transcription of the *OsGH3.8* gene and the involvement of auxin in regulating plant architecture through the miR156f/*OsSPL7* pathway. Our findings may provide a useful breeding resource for modifying rice plant architecture and enhancing rice yield.

## Materials and methods

### Plant species and growth conditions

The rice *cd* mutant was selected from a T-DNA insertion collection generated from the wild type (WT) Zhonghua 11 (*Orayza sativ*a L. subsp. *japonica* cv. Zhonghua No. 11, ZH11) (Wang *et al.*, 2004). All the rice plants were grown in a greenhouse, with 10 h light and 14 h dark, or in the field under natural conditions in summer, Shanghai, China.

### Tiller outgrowth analysis

Four-week-old rice seedlings were used for analysis; plants were peeled carefully to expose the young tiller buds in the basal region where tillers begin to develop.

### Treatment of rice plants with naphthaleneacetic acid (NAA)

Four-week-old WT rice seedlings were watered with 10 μM NAA, and samples were collected at the indicated time after treatment.

### Expression profile analysis

For expression profile analysis of miR156f, and some *OsSPL* genes, 1-month-old WT plants were used to collect leaf, leaf sheath, roots, and stems; and young panicles were collected at the reproductive stage from the WT.

### Anatomical analysis and *in situ* hybridization

Anatomical analysis and *in situ* hybridization were carried out as previously described by Wang *et al.* (2010) and Dai *et al.* (2016), respectively.

### Plasmid construction and transformation of rice

The full-length *IPS* gene of Arabidopsis was amplified by KOD-plus DNA polymerase (TOYOBO) using primers IPSF and IPSR, cloned into the PBSK vector using *Bam*HI and *Kpn*I, and the mature miRNA region was substituted into the MIM156f sequence using overlapping PCR (Higuchi *et al.*, 1988); IPSF was paired with MIM156R and IPSR with MIM156F to get the substituted sequence into two separate but overlapping PCR fragments, and then used as template. IPSF and IPSR were used as primers to amplify the full-length IPS-MIM156f fragment. The IPS-MIM156f fragment was re-cloned into p1301-35SNos vector to form the MIM156fOE vector.

*OsSPL7*, *OsSPL2*, *OsSPL13*, and *OsGH3.8* overexpression (OE) vectors were constructed by cloning full-length cDNA of respective genes into the p1301-35SNos vector using *Bam*HI and *Kpn*I double digestion.

RNAi of *OsSPL7* (SPL7RNAi) and *OsGH3.8* (GH3.8R) were constructed by cloning a 466 bp and a 273 bp cDNA fragment, respectively, into p1301RNAi vector in the sense orientation using *Bam*HI and *Kpn*I, and in the antisense orientation using *Sac*I and *Spe*I.

OsSPL7::SPL7Flag (SPL7Flag) vector was constructed by cloning the full-length cDNA of *OsSPL7* in-frame into the p1305 vector using *Kpn*I and *Bam*HI, and the *OsSPL7* promoter was further cloned using *Sac*I and *Kpn*I.

SPL7::GUS plasmid was constructed by amplifying the *OsSPL7* promoter using primers SPL7PF2 and SPL7PR and cloned into the promoter::GUS plasmid derived from pCAMBIA1301.

MIM156fOE, SPL2OE, SPL7OE, SPL13OE, SPL7RNAi, GH3.8R, SPL7::GUS, and OsSPL7::SPL7Flag plasmid were transformed into WT rice using the *Agrobacterium*-mediated method with minor modification (Hiei *et al.*, 1994).

### RT–PCR analysis and quantitative real-time RT-PCR (qRT-PCR)

Total RNA was extracted from different tissues using TRIzol (Invitrogen), followed by DNase I digestion. For reverse transcription–PCR (RT–PCR) analysis, 1 μg of total RNA was reverse transcribed using oligo(dT) primer and M-MLV reverse transcriptase (TOYOBO) according to the manufacturer’s instructions. RT–PCR analyses were performed at least three times with the *actin* gene as internal control.

For quantitative real-time PCR (qRT-PCR), cDNA was synthesized from 1 μg of total RNA using the One Step SYBR PrimeScript RT-PCR Kit (TaKaRa), and 1 μl of cDNA was used as template for real-time analysis. Sampling and expression measurement were repeated three times with the *actin* gene as internal reference.

### Yeast-one-hybrid assays

The full-length cDNAs of *OsSPL2*, *OsSPL7*, and *OsSPL13* were amplified with gene-specific primers (see Supplementary Table S1 at *JXB* online), and then fused into the activation domain (AD) in vector pPC86. Fragments containing ‘GTAC’ in the *OsGH3.8* promoter were amplified with gene-specific primers and fused into the vector p178 at the *Xho*I site. The p178 and pPC86 constructs were co-transformed into the yeast strain EGY48. The yeast was growth on SD selective medium (SD-His-Leu) and observed in blue on Chromogenic medium. The transformants containing void plasmid pPC86 and p178 constructs were used as negative control. Yeast one-hybrid assay was carried out as described (Matchmaker One-hybrid System; Clontech).

### miRNA northern blot analysis

Approximately 30 μg of total RNA was separated on 15% polyacrylamide denaturing gels. RNAs were transferred to Amersham Hybond™-N^+^ membranes and cross-linked by UV irradiation; the membranes were hybridized with biotin-labeled DNA probes complementary to the miRNA sequences at 42 °C overnight. The membranes were then washed and incubated with stabilized streptavidin–horseradish peroxidase at 42 °C. After washing with substrate equilibration buffer and adding stable peroxide solution and enhancer solution, the membranes were imaged using an FLA-5000 Phosphorimager. The DNA probes were synthesized and biotin labeled using a 3′ end DNA labeling method.

### ChIP analysis

Immunoprecipitation of DNA associated with modified histones was carried out according to the instructions of the EpiQuik™ Plant ChIP Kit. Rice young panicles were cross-linked, and Flag antibody was used for ChIP, with normal mouse IgG as the negative control. The immunoprecipitated sample and whole-cell extract (input) were incubated at 65 °C to reverse cross-linked DNA, and ethanol precipitation to elute purified DNA. ChIP DNA and whole-cell extract (input) were subjected to 35 cycles of qRT-PCR with the primers designed to amplify a sequence in the promoter, with a sequence in the coding region as the control.

### Dual luciferase (LUC) analysis

The plasmid pHB::SPL7-GFP was transformed into *Agrobacterium tumefaciens* strain GV3101 to act as an effector. The reporter construct was constructed by inserting the *OsGH3.8* promoter into the pGreenII 0800-LUC vector (Hellens *et al.*, 2005) and subsequently co-transformed with the helper plasmid pSoup19 into GV3101 to act as the reporter. pHB::GFP plasmid was used as negative control. Overnight *A. tumefaciens* cultures were collected by centrifugation, re-suspended in Murashige and Skoog (MS) medium to OD_600_=0.6, and incubated at room temperature for 3 h. The reporter strain was mixed with the effectors strain harboring pHB::SPL7-GFP or pHB::GFP at a ratio of 1:1. The mixture of *A. tumefaciens* suspension was infiltrated into tobacco (*Nicotiana benthamiana*) leaves, with the experimental group and the control group infiltrated into the opposite position on the same leaves. The leaves were collected after 3 d under long-day white light conditions and infiltrated with 150 μg ml^–1^ luciferin solution; images were captured using a CCD camera 5 min later, and quantification was performed using a Dual-Luciferase Reporter Assay System according to the instructions (Promega, Madison, WI, USA). Three biological repeats were measured for each sample.

### Histochemical GUS staining

β-Glucuronidase (GUS) staining was carried out by staining different tissues of the SPL7::*GUS* transgenic plants in GUS reaction solution which contained 100 mM sodium phosphate, 10 mM EDTA, 0.1% (v/v) Triton X-100, and 1 mM 5-bromo-4-chloro-3-indolyl-β-glucuronic acid (Sigma. USA) with pH 7.0, overnight at 37 °C, and then clearing in 75% ethanol.

### Microarray analysis

Seedlings of the 4-month old *cd* mutant and WT were used for microarray analysis. Ten plants for each sample were collected to extract total RNA and sent to the Beijing Genomics Institute for the following tests and analysis. RNA quality was checked using a NanoPhotometer^®^ spectrophotometer (IMPLEN, CA, USA). A 3 μg aliquot of total RNA per sample was used as input material for the RNA library construction with NEBNext^®^ Multiplex RNA Library Prep Set for Illumina^®^ (NEB, USA) following the manufacturer’s recommendations. After cluster generation, the library preparations were sequenced on an Illumina Hiseq2000 Platform and the clean reads were mapped to the *O. sativa* genome (RGAP, version 7.0) using cufflink software after filtering the dirty raw reads.

### Subcellular localization of OsSPL7 protein in rice protoplasts

The pA7-EGFP vector was used to construct green fluorescent protein (GFP) fusion proteins or as a negative control. The coding sequence of *OsSPL7* was cloned into pA7-EGFP to generate OsSPL7–GFP. The mCherry–VirD2NLS (mCherry) vector was used as nuclear control. The fusion constructs or control plasmids were transformed into rice protoplasts. Fluorescence was visualized under a Zeiss Axioimager Z2 fluorescence microscope. Experiments were biologically repeated three times.

### Accession numbers

*OsSPL2* (LOC_Os01g69830), *OsSPL3* (LOC_Os02g04680), *OsSPL4* (LOC_Os02g07780), *OsSPL7* (LOC_Os04g46580), *OsSPL11* (LOC_Os06g45310), *OsSPL12* (LOC_Os06g49010), *OsSPL13* (LOC_Os07g32170), *OsSPL14* (LOC_Os08g39890), *OsSPL16* (LOC_Os08g41940), *OsSPL17* (LOC_Os09g31438), *OsSPL18* (LOC_Os09g32944), *OsSPL19* (LOC_Os11g30370), *OsGH3.1* (AK063368), *OsGH3.2* (LOC-Os01g55940), *OsGH3.3* (AK072125), *OsGH3.4* (AK101932), *OsGH3.5* (AK071721), *OsGH3.6* (AK106538), *OsGH3.7* (AK099376), *OsGH3.8* (AK101193), *OsGH3.9* (AK106839), *OsGH3.10* (LOC-Os07g38860), *OsGH3.11* (LOC-Os07g47490), *OsGH3.12* (LOC-Os11g08340), *OsGH3.13* (LOC_Os11g32510), *OsGH3.14* (LOC_Os11g32520).

## Results

### A T-DNA insertion mutant with a cluster and dwarf phenotype is associated with overexpression of miR156f

**Fig. 1.**
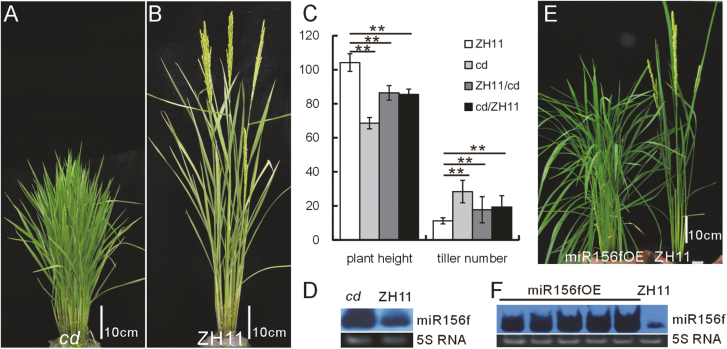
Characterization of the *cd* mutant phenotype and association of miR156f expression with the cluster and dwarf phenotype. (A and B) Representative *cd* mutant (A) and WT (B) plants. (C) Plant heights and tiller numbers of the *cd* mutant, and the crosses between the *cd* mutant and WT. The asterisks represent a significant difference, as determined by a Student’s *t*-test at *P<0.05 and **P<0.01, respectively (*n*=20). (D) miR156f expression in the *cd* mutant. (E) Phenotype of the miR156fOE transgenic plants (left) as compared with the WT (right). (F) miR156f expression in the miR156fOE lines.

We identified a T-DNA insertion mutant from our mutant population that showed an obvious cluster (increased number of tillers) and a dwarf (decreased plant height) phenotype (Wang *et al.*, 2004), and named it *cd* (*cd* is shown in Fig. 1A and compared with the WT in Fig. 1B). Under natural field conditions in summer in Shanghai, the *cd* mutant had an average plant height of 62.7 cm, in comparison with the WT plants which were on average 94.9 cm tall. The *cd* mutant had an average of 42.1 effective tillers, while the WT plants had 14.1 (Fig. 1C). The *cd* mutant also flowered ~5–6 d later than the WT plants (data not shown).

We next carried out genetic analysis by making genetic reciprocal crosses of the *cd* mutant with the WT. The F_1_ plants from both the reciprocal cross and backcross had semi-cluster and semi-dwarf phenotypes (Fig. 1C). This suggests that the phenotype of the *cd* mutant is semi-dominant. Among the 691 F_2_ population of the *cd*/ZH11 cross, there were 170 taller plants, 352 average height plants, and 169 dwarf plants. The average height plants and dwarf plants were resistant to hygromycin. Among the F_2_ population of the ZH11/*cd* cross, the ratio of taller to average height to dwarf plants was 211:392:189. Therefore, the segregation ratios of both the reciprocal cross and the backcross were ~1:2:1, which is in accordance with Mendelian segregation. Furthermore, in the F_2_ population, both the cluster and dwarf phenotypes showed tight co-segregation with the T-DNA insert (Supplementary Table S2). From our results, we concluded that the cluster and dwarf phenotype in the *cd* mutant was controlled by a single semi-dominant genetic locus.

Next, we isolated the T-DNA flanking sequence in the *cd* mutant using thermal asymmetric interlaced PCR (TAIL-PCR) (Liu *et al.*, 1995). This revealed that the T-DNA was integrated at position 21575719 of NC-008401.2 (http://www.ncbi.nlm.nih.gov/, accessed 27 July 2018), and that the *miR156f* gene is positioned ~9 kb upstream of the insertion. miRNA northern blot analysis showed that miR156f was markedly up-regulated in the *cd* mutant (Fig. 1D).

To test whether up-regulation of miR156 caused the cluster and dwarf phenotype, we cloned the *miR156f* gene and genetically overexpressed it. More than 90% of the transgenic plants (miR156fOE) had a cluster and dwarf phenotype that was similar to that of the *cd* mutant (Fig. 1E). We sampled five T_0_ plants and confirmed miR156f overexpression in all of them (Fig. 1F). Together, these results confirm that the T-DNA insertion in the *cd* mutant causes up-regulation of miR156f, which leads to the cluster and dwarf phenotype.

### Plants overexpressing an miR156f target mimic (MIM156fOE) have fewer tillers and increased plant height

To better understand the function of miR156f in rice, we constructed a target mimic of miR156f (MIM156f) and genetically overexpressed it in WT plants (Franco-Zorrilla *et al.*, 2007).

**Fig. 2.**
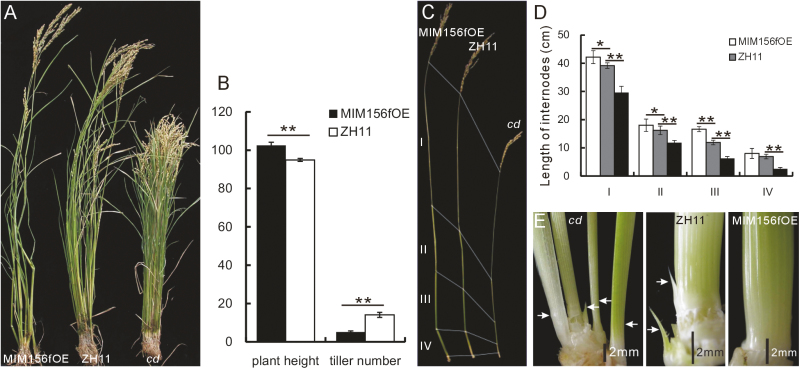
Phenotype of the MIM156f OE plants. (A) The appearances of the MIM156fOE, WT, and *cd* mutant plants (left to right). (B) The plant heights and tiller numbers of the MIM156fOE plants in comparison with the WT plants (*n*=20). (C) Appearance of the stems of the MIM156fOE, WT, and *cd* mutant plants, showing the numbered internodes I, II, III, IV. (D) Lengths of each internode in the MIM156fOE, WT, and *cd* mutant plants (*n*=10). The asterisks in (B) and (D) represent a significant difference, as determined by a Student’s *t*-test at *P<0.05 and **P<0.01, respectively. (E) The basal region of the stems of the *cd* mutant, WT, and MIM156fOE plants (left to right).

Approximately 91.2% of the transgenic plants (MIM156fOE) had a reduced number of tillers and an increased plant height (Fig. 2A). MIM156f overexpression was confirmed in the transgenic plants, while endogenous miR156f was down-regulated (Supplementary Fig. S1A). Expression of *OsSPL13*, a candidate target of miR156, was up-regulated (Supplementary Fig. S1A). These results suggest that MIM156f expression interferes with the expression of endogenous miR156 and its targets.

The T_1_ generation of MIM156fOE plants were grown in the field and analyzed. The MIM156fOE plants had an average of 5.1 tillers, in comparison with 14.1 tillers in the WT plants. The MIM156fOE plants had an average height of 102.5 cm, while the average height of the WT plants was 94.9 cm (Fig. 2B). Further analysis revealed that miR156 expression influenced the elongation of the internodes in rice plants. The length of almost every internode was increased in the MIM156fOE plants, while it was reduced in the *cd* mutant. These changes in internode length influenced the overall plant height accordingly (Fig. 2C, D).

Development of tillers in rice involves two successive processes: the formation of an axillary bud at the basal region of the stem, and subsequent outgrowth. A defect in either process can influence the number of tillers (Li *et al.*, 2003). We examined the role of miR156f at the tiller initiation stage, and found that there were fewer axillary buds at the basal region of the stem in young MIM156fOE plants, in comparison with the WT (Fig. 2E). In contrast, there were more axillary buds in the *cd* mutant in comparison with the WT (Fig. 2E). These data suggest that miR156f regulates the initiation of the axillary buds. Our combined data show that miR156f regulates tiller number and plant height in rice.

### Transgenic plants overexpressing *OsSPL7* have a single tiller and *OsSPL7* RNAi plants have a cluster and dwarf phenotype

miRNAs function mainly through negative regulation on their targets. In rice, there are 12 and 18 genes coding for miR156 and its target *OsSPL* genes, respectively (http://www.mirbase.org, accessed 27 July 2018; Xie *et al.*, 2006). In the microarray analysis of the *cd* mutant, most of the *OsSPL* genes were down-regulated. Among these genes, *OsSPL2*, *OsSPL7*, and *OsSPL13* were the most down-regulated, according to the fold change (Supplementary Table S3). We examined *OsSPL* expression in the *cd* mutant, miR156fOE, and MIM156fOE plants using qRT-PCR, and further confirmed the regulatory effect of miR156f on the expression of most *OsSPL* genes (Supplementary Fig. S1B). To examine miR156f function further, we carried out genetic analysis of *OsSPL2*, *OsSPL7*, and *OsSPL13*.

**Fig. 3.**
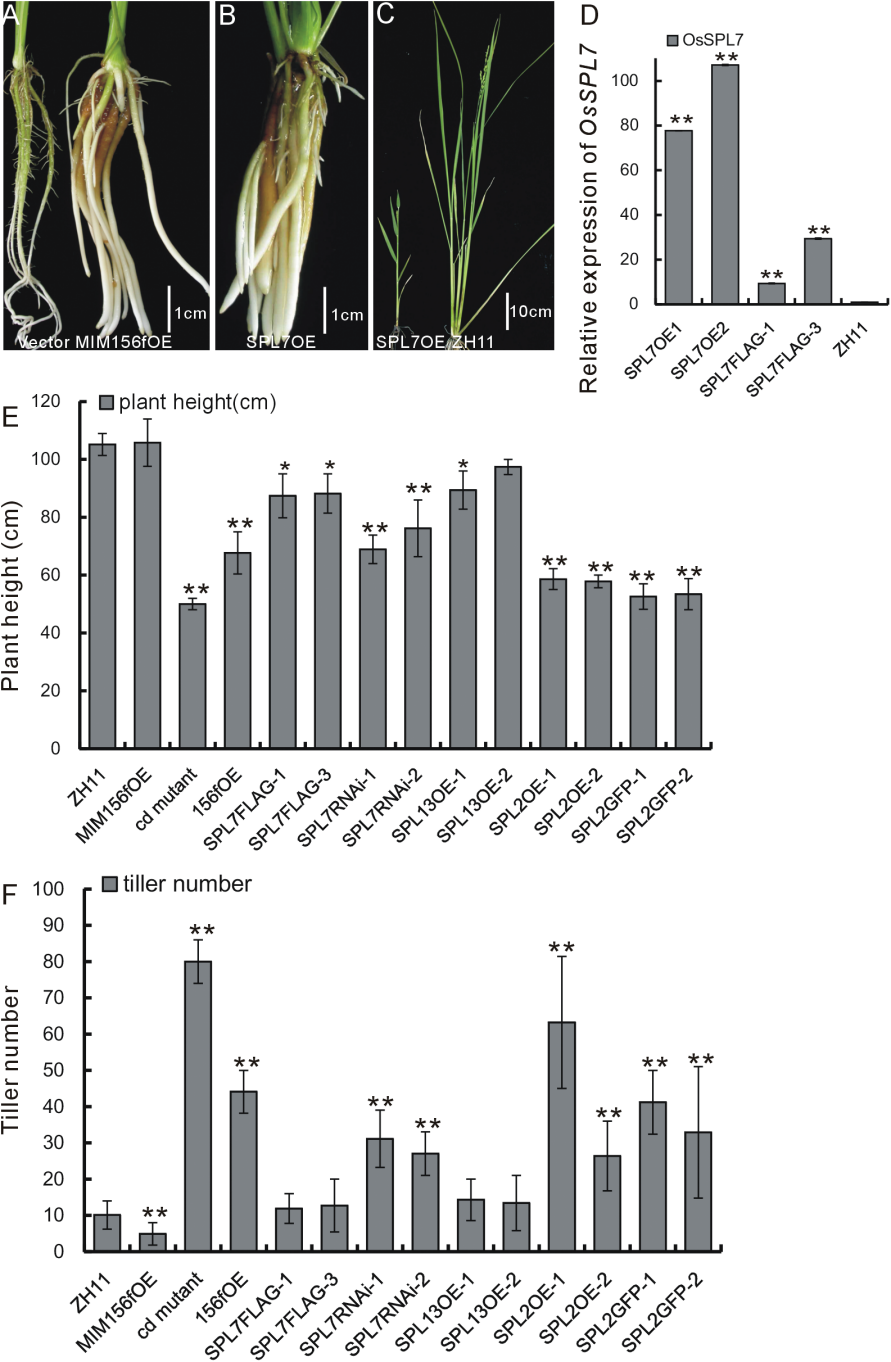
Expression of *OsSPL* genes and functional activity. (A) Root phenotype of a representative T_0_ generation MIM156fOE plant in a tissue culture tube (control phenotype of the transgenic plant from the empty vector is shown on the left for comparison). (B) Root phenotype of a representative T_0_ generation SPL7OE plant in a tissue culture tube. (C) Appearance of a representative SPL7OE plant in comparison with a WT plant. (D) Relative expression levels of *OsSPL7* in SPL7OE and SPL7Flag plants (*n*=3). (E) Heights of the miR156- and *OsSPL* -related mutant and transgenic plants (*n*=20). (F) Tiller numbers of the miR156- and *OsSPL*-related mutant and transgenic plants (*n*=20). The asterisks in (D–F) represent a significant difference, as determined by a Student’s *t*-test at *P<0.05 and **P<0.01, respectively.

First, we individually overexpressed each of these three genes in the WT background. Transgenic plants overexpressing *OsSPL7* (SPL7OE) had thickened roots with fewer lateral roots (Fig. 3B), and resembled the MIM156fOE phenotype (Fig. 3A). Cellular analysis revealed that the thickened roots in the MIM56fOE plants were associated with an increased number of parenchymal cells (Supplementary Fig. S2A–C). Furthermore, most SPL7OE plants had single tillers and were short and infertile (Fig. 3C). To rule out the possibility that the infertility of the SPL7OE plants might be caused by high-level expression of *OsSPL7* under the 35S promoter, we generated SPL7Flag transgenic lines with *OsSPL7* under its native promoter and fused to a Flag tag. In these SPL7Flag plants, the *OsSPL7* gene was still up-regulated, but to a lesser degree than in the SPL7OE plants (Fig. 3D). Moreover, the phenotype of the SPL7Flag plants was not as severe as that of the SPL7OE plants. The SPL7Flag plants had a reduced height in comparison with the WT (Fig. 3E, F), but a similar tiller number. We also constructed *OsSPL7* RNAi transgenic plants (SPL7RNAi), which had reduced plant heights and increased tiller numbers, and resembled the *cd* mutant and the miR156fOE plants (Fig. 3E, F).

**Fig. 4.**
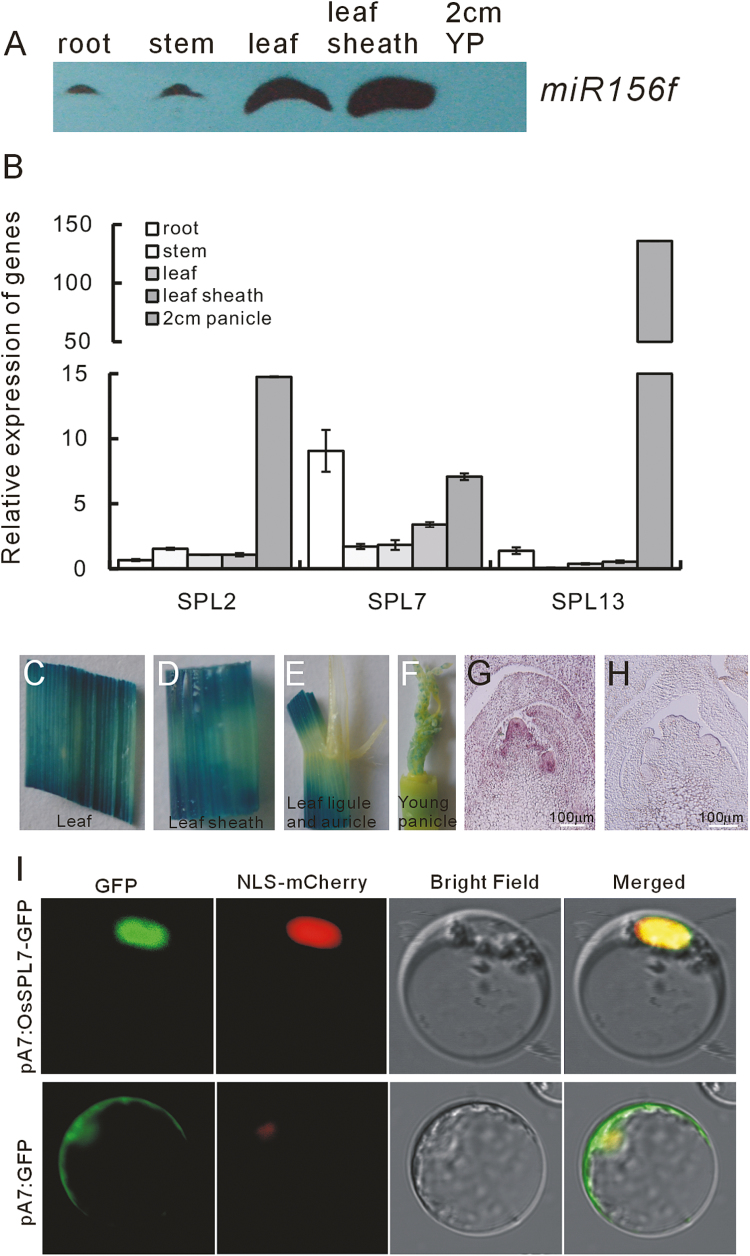
miR156f and *OsSPL7* expression profiles and localization of the OsSPL7–GFP fusion protein. (A) Northern blot analysis of miR156f in different tissues (30 μg of total RNA loaded for each). YP, young panicle. (B) Expression of *OsSPL2*, *OsSPL7*, and *OsSPL13* in different tissues (the same samples as in A) as revealed by qRT-PCR (*n*=3). (C–F) GUS staining of the SPL7::GUS transgenic plant tissues, namely the leaf (C), the leaf sheath (D), the leaf ligule and auticle (E), and the 1.5 cm panicle (F). (G) *In situ* hybridization of *OsSPL7* mRNA at the panicle initiation stage. (H) Sense probe signal from the *in situ* hybridization. (I) Localization of the OsSPL7–GFP fusion protein in the rice protoplast.

To characterize *OsSPL7* further, we analyzed its expression profile together with that of miR156f (Fig. 4A). miR156f was expressed at low levels in the roots and very young panicles (2 cm in length). However, there was clear *OsSPL7* expression in the root (Fig. 4B), in accordance with its function in root development (Fig. 3B). In young panicles, *OsSPL7* showed a relatively high expression level (Fig. 4B). In comparison, expression of *OsSPL2* and *OsSPL13* was not as high in the roots, and, similarly, both *OsSPL2* and *OsSPL13* showed high expression in the 2 cm panicle whereas miR156f was undetectable (Fig. 4B). We also constructed SPL7::GUS transgenic plants in which the *OsSPL7* promoter was used to drive *GUS* gene expression. The GUS signal was detected in the leaf and leaf sheath (Fig. 4C, D), and the young 1.5 cm panicle (Fig. 4F), but not the leaf ligule or the leaf auricle (Fig. 4E). *In situ* hybridization confirmed the *OsSPL7* expression at the early stage of panicle development (Fig. 4G). Together, these results suggest that OsSPL7 is a ubiquitous protein.

We also examined OsSPL7–GFP fusion protein expression in rice protoplasts and found that the florescence signal was exclusively concentrated in the nucleus (Fig. 4I).

Plants overexpressing *OsSPL13* (SPL13OE) had a similar phenotype to that of SPL7Flag plants, while plants overexpressing *OsSPL2* alone (SPL2OE) or tagged with GFP (SPL2GFP) both had reduced plant height and increased tiller numbers, and resembled the *cd* mutant (Fig. 3E, F).

These genetic analyses suggest that several *OsSPL* genes might be involved in the regulation of plant architecture, but with differing degrees of activity and function. In this study, we chose to focus on *OsSPL7* as the downstream target of miR156 due to its phenotypic similarity to miR156f genetic plants.

We also found that there was up-regulation of miR156f in SPL2OE, SPL7OE, and SPL13OE plants, indicating that increased *OsSPL* expression has feedback regulation effects on miR156f (Supplementary Fig. S1C).

### *OsGH3.8* is directly regulated by OsSPL7

The role of auxin in maintaining apical dominance by suppressing axillary development is important. The *cd* mutant developed more ectopic shoots, while there were fewer in the MIM156fOE plants. Moreover, in the *cd* mutant, the dormancy of axillary buds was disrupted, especially in the upper nodes (Supplementary Fig. S2D). The disturbed axillary bud dormancy and ectopic shoots in the *cd* mutant suggested the possible involvement of auxin in miR156f function. To investigate this, we examined the *cd* mutant microarray data for auxin factors and found that *OsGH3.8* was up-regulated among the *OsGH3* family genes (Supplementary Table S3). Further, qRT-PCR analysis confirmed that *OsGH3.8* was the most significantly up-regulated of these genes in the *cd* mutant (Supplementary Fig. S1D).

**Fig. 5.**
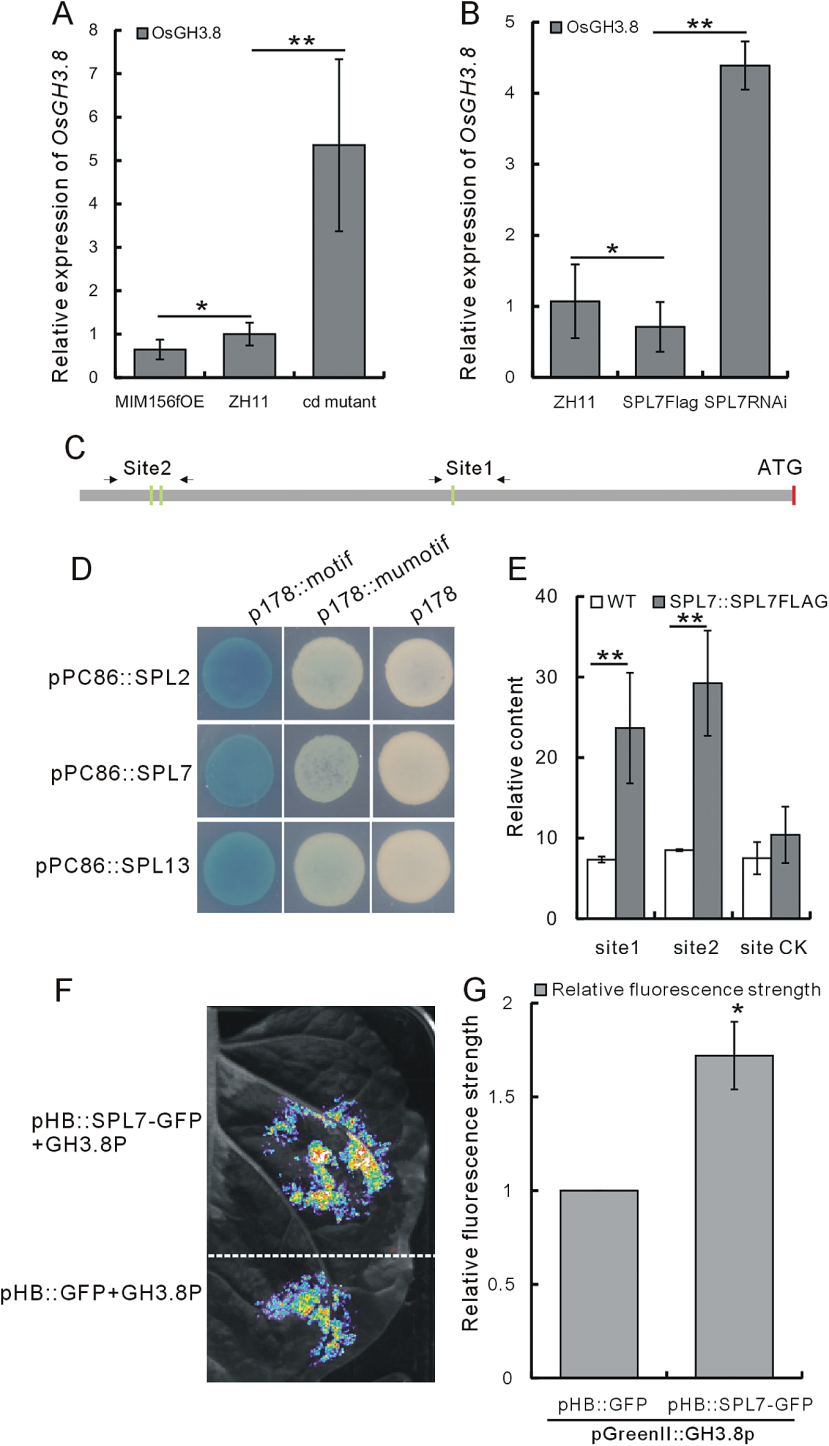
Confirmation of the transcriptional regulation of the *OsGH3.8* gene by OsSPL7 protein. (A) *OsGH3.8* expression in the *cd* mutant and the MIM156fOE plants (*n*=3). (B) *OsGH3.8* expression in the SPL7Flag and SPL7RNAi plants (*n*=3). (C) Schematic of the SPL-binding motifs in the 2 kb *OsGH3.8* promoter. Green bars indicate the positions of the GTAC motifs at 1022 bp (site 1), 1932 bp, and 1936 bp motifs (site 2). The regions amplified by the correspondingly named primers (see the Materials and methods) at sites 1 and 2 are indicated by arrows. (D) Yeast one-hybrid analysis of the OsSPL2, OsSPL7, and OsSPL13 proteins binding to the 1022 bp motif. (E) ChIP analysis of SPL7Flag binding to sites 1 and 2 (*n*=3). (F) Image of the dual-LUC assay. (G) The LUC/REN ratio in the dual-LUC assay indicating relative luciferase activity. The empty vector pHB::GFP was used as control. Values are given as the mean±SD (*n*=3). The asterisks in (A), (B), (E), and (G) represent a significant difference, as determined by a Student’s *t*-test at *P<0.05 and **P<0.01, respectively.

We further measured *OsGH3.8* expression in the *cd* mutant, the WT, and MIM156fOE plants, and found that *OsGH3.8* was up-regulated in the *cd* mutant and down-regulated in MIM156fOE plants (Fig. 5A; Supplementary Fig. S3A). Moreover, *OsGH3.8* was down-regulated in SPL7Flag plants and up-regulated in SPL7RNAi plants (Fig. 5B; Supplementary Fig. S3B). These data suggest that miR156f/*OsSPL7* might modulate plant architecture by regulating *OsGH3.8*.

Next, we examined whether there were any SPL-binding motifs in the 2 kb *OsGH3.8* promoter (http://www.dna.affrc.go.jp/PLACE/signalscan.html). We identified three GTAC motifs, located 1022, 1932, and 1936 bp upstream of the ATG initiation codon (Fig. 5C). Using a yeast one-hybrid approach, we found that OsSPL7 could bind to the 1022 bp motif. When the GTAC motif was mutated to GAAC, OsSPL7 could no longer bind to it (Fig. 5D), indicating that OsSPL7 specifically binds to the *OsGH3.8* promoter through the GTAC motif. Moreover, our yeast one-hybrid analysis also showed that OsSPL2 and OsSPL13 could bind to the 1022 bp GTAC motif (Fig. 5D). These data suggest that at least some SPL proteins can bind to the *OsGH3.8* promoter.

ChIP analysis of the SPL7Flag plants using a Flag antibody showed obvious binding to the sites corresponding to the 1022 bp motif (site1; Fig. 5E) and the combined 1932 bp and 2426 bp motifs (site2; Fig. 5E). Furthermore, to validate the activation of the *OsGH3.8* gene by OsSPL7, we carried out a dual-LUC assay in tobacco leaf; it was revealed that OsSPL7 activated the expression of the *OsGH3.8* gene promoter which showed a higher value of LUC/REN than the GFP control (Fig. 5F, G).

From our combined analyses, we conclude that OsSPL7 can directly bind to the *OsGH3.8* promoter and regulate its expression.

### Overexpression of *OsGH3.8* partially complements the MIM156fOE phenotype

**Fig. 6.**
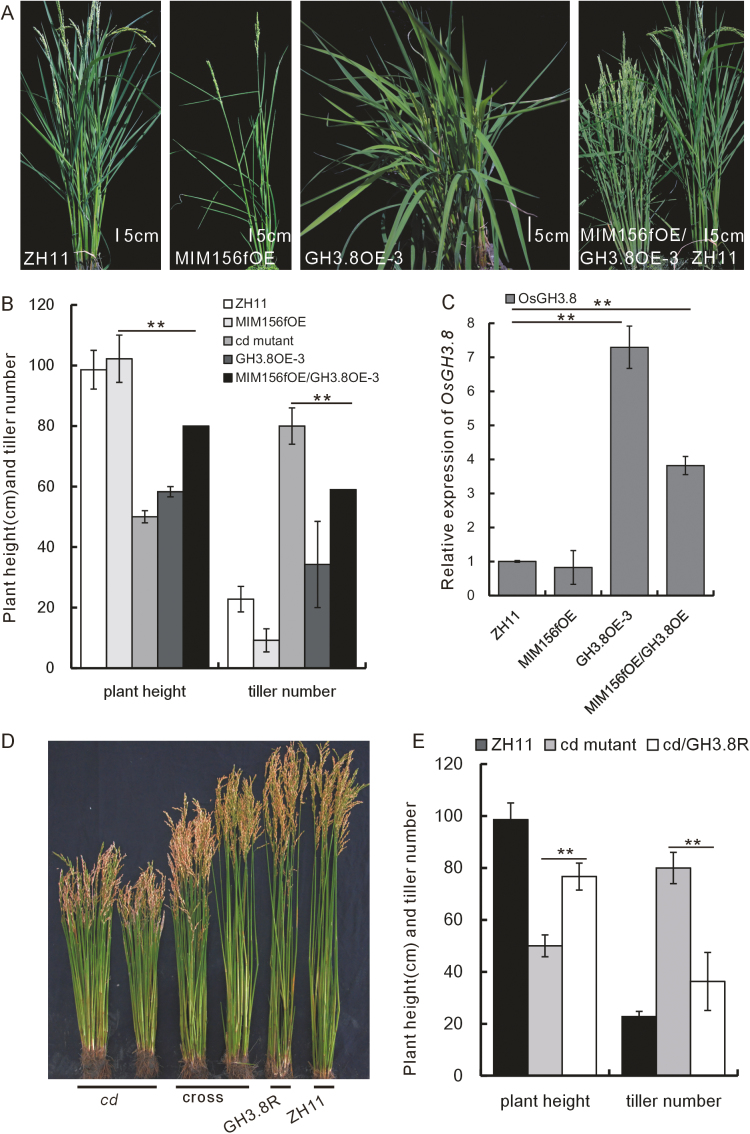
Characterization of MIM156fOE/GH3.8OE and *cd*/GH3.8R hybrid plants. (A) Appearance of representative WT, MIM156fOE, GH3.8OE-3, and hybrid MIM156fOE/GH3.8OE-3 plants (left to right). (B) Plant height and tiller number of the MIM156fOE/GH3.8OE-3 hybrid and genetically related plants (*n*=20). (C) *OsGH3.8* expression in the MIM156fOE/GH3.8OE-3 plant and its parent lines (*n*=3). (D) Plant height and tiller number phenotypes of the *cd* mutant, the *cd*/GH3.8R hybrid, and the GH3.8R and WT plants (left to right). (E) Plant height and tiller number of the *cd*/GH3.8R hybrid as compared with the *cd* mutant and WT (*n*=10). The asterisks in (B), (C), and (E) represent a significant difference, as determined by a Student’s *t*-test at *P<0.05 and **P<0.01, respectively.

To characterize further the genetic relationship between *OsGH3.8* and miR156f, we cloned the *OsGH3.8* gene and overexpressed it in the WT background. The transgenic plants (OsGH3.8OE) showed up-regulation of *OsGH3.8*, an increased number of tillers, and a reduced plant height in comparison with the WT (Fig. 6A).

We next crossed the OsGH3.8OE-3 and OsGH3.8OE-4 transgenic plant lines with homozygous MIM156fOE plants. We obtained a single MIM156f/GH3.8OE-3 hybrid plant and two MIM156f/GH3.8OE-4 hybrid plants. We molecularly confirmed these genotypes by the existence of the plasmids used for transformation (Supplementary Fig. S3C). The hybrid plants had an increased number of tillers and a reduced plant height, in comparison with MIM156fOE, with phenotypes intermediate between those of MIM156fOE and *cd* mutant plants (Fig. 6A, B). *OsGH3.8* was also up-regulated in the hybrid plants (Fig. 6C).

 Next, we constructed OsGH3.8RNAi transgenic plants (OsGH3.8R). Although they did not show obvious phenotypic changes in tiller number or plant height (Fig. 6D), when we crossed the OsGH3.8R plants with the *cd* mutant, the hybrid had obvious changes in both features that were intermediate between those of the WT and the *cd* mutant (Fig. 6D, E).

From these hybridizations, we found that *OsGH3.8* overexpression complemented the architectural defects of MIM156fOE plants by increasing the tiller number and reducing the plant height; whereas RNAi of *OsGH3.8* in the *cd* mutant complemented the *cd* mutant phenotype. The direct binding of OsSPL7 to the *OsGH3.8* promoter and the genetic relationship between miR156f and *OsGH3.8* suggest that miR156f/*OsSPL7* could directly regulate *OsGH3.8* to modulate plant architecture.

**Fig. 7.**
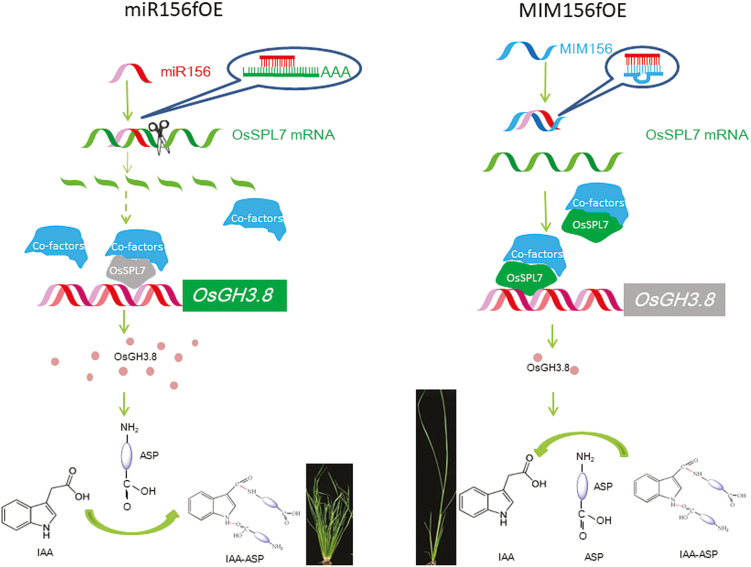
Schematic of the proposed miR156–OsSPL7–OsGH3.8 pathway. In miR156fOE plants (left), excessive miR156 cleaves *OsSPL7* transcripts and represses its expression. This releases OsSPL7 transcriptional control of *OsGH3.8* expression. The plentiful OsGH3.8 facilitates the combination of IAA with aspartic acid (ASP), leading to cluster and dwarf plant phenotypes. In MIM156fOE plants, miR156f is sequestered by MIM156f, so that there is enough OsSPL7 available to suppress the expression of *OsGH3.8*. This then promotes the dissociation of ASP from IAA, leading to a phenotype of reduced tiller number and increased plant height.

Together, we propose the following model (Fig. 7). First, miR156f negatively regulates its target *OsSPL7*, which turns out to be a negative regulator of *OsGH3.8*. *OsGH3.8* promotes the combination of IAA with aspartic acid, and the consequent change in IAA content influences plant architecture to alter the plant height and tiller number in rice. Since *OsGH3.8* is a fast-acting auxin-responsive gene, treatment of rice plants with NAA could rapidly induce the expression of *OsGH3.8*. This might have a feedback effect to reduce miR156f expression (Supplementary Fig. S4A, B).

### The involvement of auxin in MIM156fOE plants might favor the grain-filling process in dense-panicle plants

**Fig. 8.**
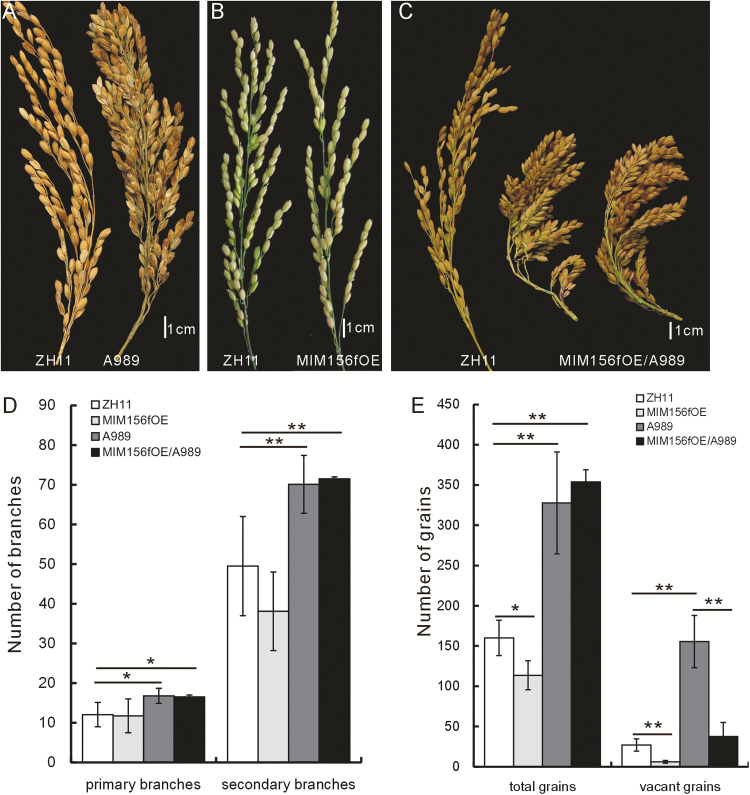
MIM156fOE favored the grain-filling process in dense-panicle plants. (A–C) Panicles of A989 (A), MIM156fOE (B), and MIM156fOE/A989(C) plants (the WT is shown to the left in each image for comparison). (D) Numbers of primary and secondary branches in the MIM156f/A989 hybrid, its parent lines, and the WT (*n*=10). (E) Numbers of total and vacant grains in the MIM156fOE/A989 hybrid, its parent lines, and the WT (n=20). The asterisks in (D) and (E) represent a significant difference, as determined by a Student’s *t*-test at *P<0.05 and **P<0.01, respectively.

There is a great challenge to improve crop plant yield to provide enough food for the increasing world population. In a previous study, we isolated a dense-panicle T-DNA insertion mutant, A989, in which the *RCN2* gene was up-regulated (Nakagawa *et al.*, 2002; Li *et al.*, 2010). The seed setting per panicle in this mutant is approximately double that of the WT; however, approximately half of the seeds are empty. Therefore, the enhanced seed set in A989 is offset by the high ratio of vacant seeds (Fig. 8A). To improve the rice yield, the filling efficiency in the A989 mutant would need to be improved.

Because auxin signaling participates in the grain-filling process in rice (Liu *et al.*, 2015), we investigated whether the miR156–OsSPL7–OsGH3.8 pathway might influence grain filing. To this end, we crossed the MIM156fOE plants with the A989 mutant. The hybrid had a similar number of tillers to that of the MIM156fOE plants, but the panicle was shortened and the seeds were densely set (Fig. 8A–C). However, the hybrid panicle had similar numbers of first and secondary branches as that of the A989 mutant (Fig. 8D). More importantly, the seed-filling efficiency of the hybrid was dramatically improved (Fig. 8E). These results show that the MIM156fOE genetic background improves the grain-filling limitation of A989.

## Discussion

It has been extensively reported that the function of miR156 is far ranging and conserved among different species (Huijser and Schmid, 2011; Wang and Wang, 2015). In this study, we showed that miR156f is a positive regulator of tiller number and a negative regulator of plant height. Consistent with its conserved function, several of the 12 *miR156* genes in rice have been reported as influencing tiller number and plant height (Xie *et al.*, 2006; Chen *et al.*, 2015; Hayashi-Tsugane *et al.*, 2015; Wang *et al.*, 2015). There are also 18 miR156 target *SPL* genes in rice, and the functions of some of these have already been determined (Jiao *et al.*, 2010; Miura *et al.*, 2010; Springer, 2010). We found that SPL7OE plants had a single tiller phenotype that has not previously been reported for other *SPL* genes (Fig. 3C). We also found that SPL7RNAi plants had a cluster and dwarf phenotype (Fig. 3E, F). Based on the concept that miRNAs negatively regulate expression of their targets, we determined that OsSPL7 is a negative regulator of tiller number and *OsSPL7* functions as a downstream target of miR156f in regulating plant architecture (Vaucheret, 2006; Liu, 2008). At the same time, *OsSPL2* functions as a positive regulator of tiller number and a negative regulator of plant height, as confirmed by the cluster and dwarf phenotype of OsSPL2OE and OsSPL2GFP plants (Fig. 3E, F; Supplementary Fig. S5A). Therefore, it appears that the targets of miR156 might function in the same processes, but the direction of regulation might differ. This might be a buffering mechanism to offset the cumulative effects of there being several targets of miR156. Supporting this hypothesis, we found that SPL7OE plants had a single tiller and were infertile (Fig. 3C), which is a phenotype that is more severe than that of the MIM156fOE plants (Fig. 2A).

In this study, we showed that there was direct binding of OsSPL7 to the *OsGH3.8* promoter. From these data, together with genetic analysis of the interaction between miR156f and *OsGH3.8*, we demonstrated that the miR156f–*OsSPL7*–*OsGH3.8* pathway is important in regulating rice plant architecture (Fig. 7). We also found that *miR156f* overexpression caused a cluster and dwarf phenotype (Fig. 1), the development of ectopic shoots, and disturbed axillary bud dormancy (Supplementary Fig. S2D). These features are associated with alterations in auxin signaling. However, our analysis did not rule out the possible involvement of other targets (such as *OsSPL2*) and additional factors that could regulate plant architecture. Based on the following observations, we propose that there might also be additional pathways through which miR156f is involved in auxin signaling in addition to the miR156f–*OsSPL7*–*OsGH3.8* pathway. First, the tiller number and plant height of the *cd* mutant were partially complemented by OsGH3.8R plants (Fig. 6D, E). Secondly, although the SPL7RNAi and GH3.8OE plants both had increased numbers of tillers and reduced plant heights, neither of these plant lines had phenotypes that were as pronounced as those in the *cd* mutant (Figs 3E, F, 6B; Supplementary Fig. S5B).

In this study, the *OsSPL7* activity had obvious dosage effects. These were associated not only with the tiller number but also with fertility, with high *OsSPL7* expression in SPL7OE plants resulting in infertility (Fig. 3C, D). In the SPL7Flag plants and the MIM156fOE plants where the *OsSPL7* levels were not as highly up-regulated (Fig. 3D; Supplementary Fig. S1B), the grain-filling efficiency and fertility were restored. Furthermore, the low grain-filling efficiency of the A989 mutant was greatly enhanced by hybridization with the MIM156fOE plant line. Therefore, both miR156f and *OsSPL7* might have roles in regulating fertility. This hypothesis is supported by previous discussions of the possible roles of the miR156/SPL in fertility regulation in Arabidopsis (Huijser and Schmid, 2011) and rice (Wang and Wang, 2015), and further extends the possible roles of miR156. Auxin is also a multifunctional factor in plant development. The combined actions of miR156 and auxin might also mediate changes in fertility such as that demonstrated by the crossing of MIM156fOE and A989 (Fig. 8). Therefore, crosstalk between important developmental and physiological factors such as miR156f and auxin might be important for modulating plant development and physiology in more than one aspect of plant growth.

## Supplementary data

Supplementary data are available at *JXB* online.

Table S1. Primer sequences used in this study.

Table S2. Co-segregation analysis of the T-DNA insert and the cluster and dwarf phenotype in the F_2_ population obtained from the reciprocal cross and backcross between WT and the *cd* mutant.

Table S3. Microarray analysis of the *cd* mutant as compared with the WT.

Fig S1. Gene expression in the miR156f-related plant lines.

Fig S2. Further phenotypes of MIM156fOE and the *cd* mutant.

Fig S3. *OsGH3.8* expression in miR156f/*OsSPL7*-related genetic lines and confirmation of the cross between the GH3.8OE and MIM156fOE plants.

Fig S4. miR156f and *OsGH3.8* expression in response to NAA treatment.

Fig S5. qRT-PCR analysis of *OsSPL2* expression in the OsSPL2OE and OsSPL2GFP plants (A) and statistical analysis of the plant height and tiller number in the *cd* mutant, SPL7RNAi and GH3.8OE plants (B).

Supplementary Figures S1-S5

Supplementary Table S1

Supplementary Table S2

Supplementary Table S3

## Abbreviations:

MIM156f: mimicry miR156f
MIM156fOE: mimicry miR156f overexpression
miR156fOE: miR156f overexpression
OsGH3.8OE: *OsGH3.8* overexpression
OsGH3.8RNAi: *OsGH3.8* RNA inference
OsGH3.8R: *OsGH3.8* RNA inference transgenic plants
SPL7Flag: *OsSPL7* fused with the Flag tag driven by its own promoter
SPL2GFP: *OsSPL2* fused with GFP driven by its own promoter
SPL7OE: *OsSPL7* overexpression
SPL2OE: *OsSPL2* overexpression
SPL13OE: *OsSPL13* overexpression
SPL7RNAi: *OsSPL7* RNA interference
smRNA: small RNA
SPL: *SQUAMOSA PROMOTER BINDING PROTEIN-like*
