# Supplementary material for: Modulation of plant architecture by the miR156f–OsSPL7–OsGH3.8 pathway in rice
Source: J Exp Bot. 2018 Jul 25;69(21):5117–30. doi: 10.1093/jxb/ery273 (PMC6184515; doi:10.1093/jxb/ery273)
Supplement: Supplementary Figures S1-S5 [file ery273_suppl_supplementary_figures_s1-s5.pdf]

Figure legends to the Supplemental Figures

Figure S1 Expression analysis of genes in miR156f related genetic lines

A, Expression analysis of *miR156f*, MIM156f and *OsSPL13* in the MIM156fOE plants; B, Expression analysis of the *OsSPL* genes in the *cd* mutant, the miR156fOE, the MIM156fOE and ZH11 plants; C, Expression analysis of *miR156f* and *OsGH3.8* in the OsSPL13OE, OsSPL7OE, OsSPL2OE and OsSPL2GFP plants; D, Expression analysis of *OsGH3* genes in the *cd* mutant. Single and double asterisks in B and D represent significant difference determined by the Student's *t*-test at $^*P<0.05$ and $^{**}P<0.01$ respectively.

Figure S2 Other phenotypes of MIM156fOE and *cd* mutant.

A, Root phenotype of the MIM156fOE plants in germination; B, Section of the root of WT ZH11; C, Section of the root of the MIM156fOE plants; D, Comparison of the status of the axillary buds in the upper nodes in the *cd* mutant and the WT ZH11.

Figure S3 Expression of *OsGH3.8* in miR156/SPL7-related genetic lines and molecular check of the cross of GH3.8OE and MIM156fOE plants

A, Expression analysis of *OsGH3.8* in MIM156fOE plants and the *cd* mutant; B, Expression analysis of *OsGH3.8 and OsSPL7* in the SPL7RNAi plants; C, Molecular check of the cross of GH3.8OE and MIM156fOE plants.

Figure S4 Expression analysis of miR156 (A) and *OsGH3.8* (B) in response to NAA treatment.

Figure S5 qRT-PCR analysis of *OsSPL2* expression in the OsSPL2OE and OsSPL2GFP plants (A) and statistical analysis of the plant height and tiller number in the *cd* mutant, the SPL7RNAi and the GH3.8OE plants (B). Single and double asterisks represent significant difference determined by the Student's *t*-test at $^*P<0.05$ and $^{**}P<0.01$ respectively.

Figure S1

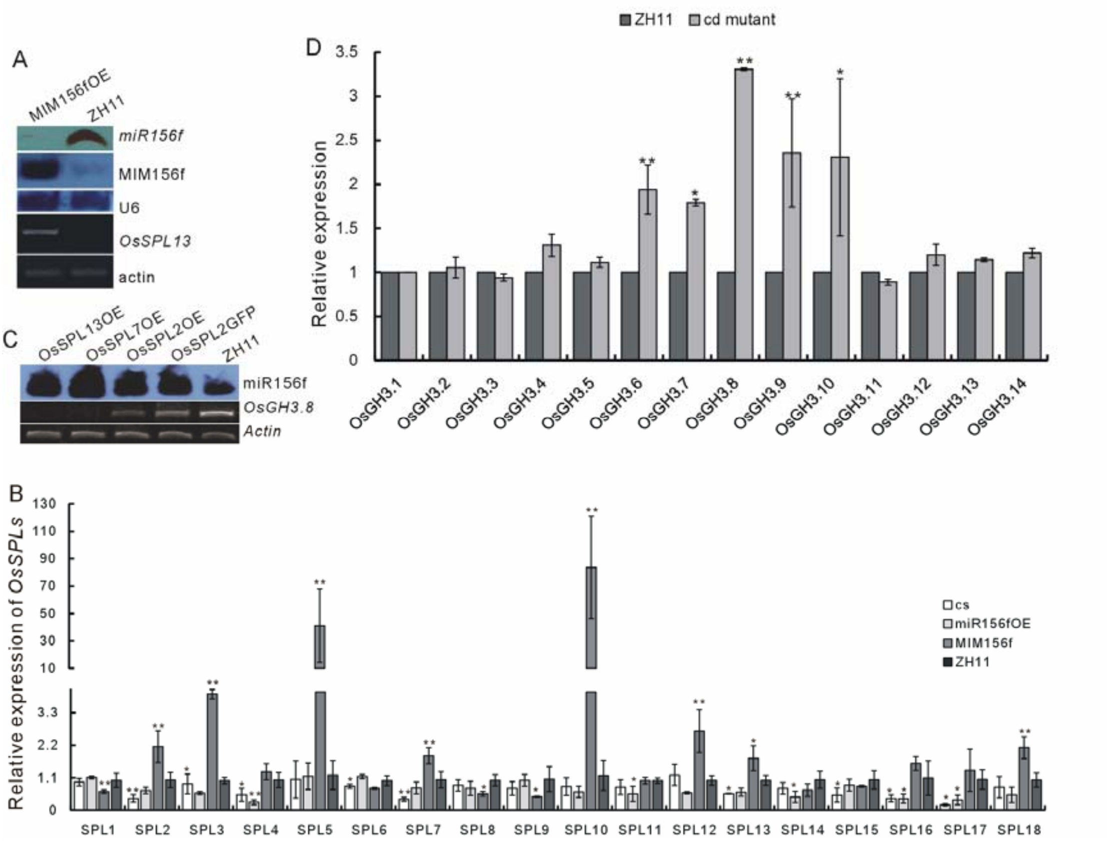
A
MIM156fOE
ZH11
miR156f
MIM156f
U6
OsSPL13
actin
C
OsSPL13OE
OsSPL7OE
OsSPL2OE
OsSPL2GFP
ZH11
miR156f
OsGH3.8
Actin
D
ZH11
cd mutant
Relative expression
OsGH3.1
OsGH3.2
OsGH3.3
OsGH3.4
OsGH3.5
OsGH3.6
OsGH3.7
OsGH3.8
OsGH3.9
OsGH3.10
OsGH3.11
OsGH3.12
OsGH3.13
OsGH3.14
B
Relative expression of OsSPLs
cs
miR156fOE
MIM156f
ZH11
SPL1
SPL2
SPL3
SPL4
SPL5
SPL6
SPL7
SPL8
SPL9
SPL10
SPL11
SPL12
SPL13
SPL14
SPL15
SPL16
SPL17
SPL18

Figure S2

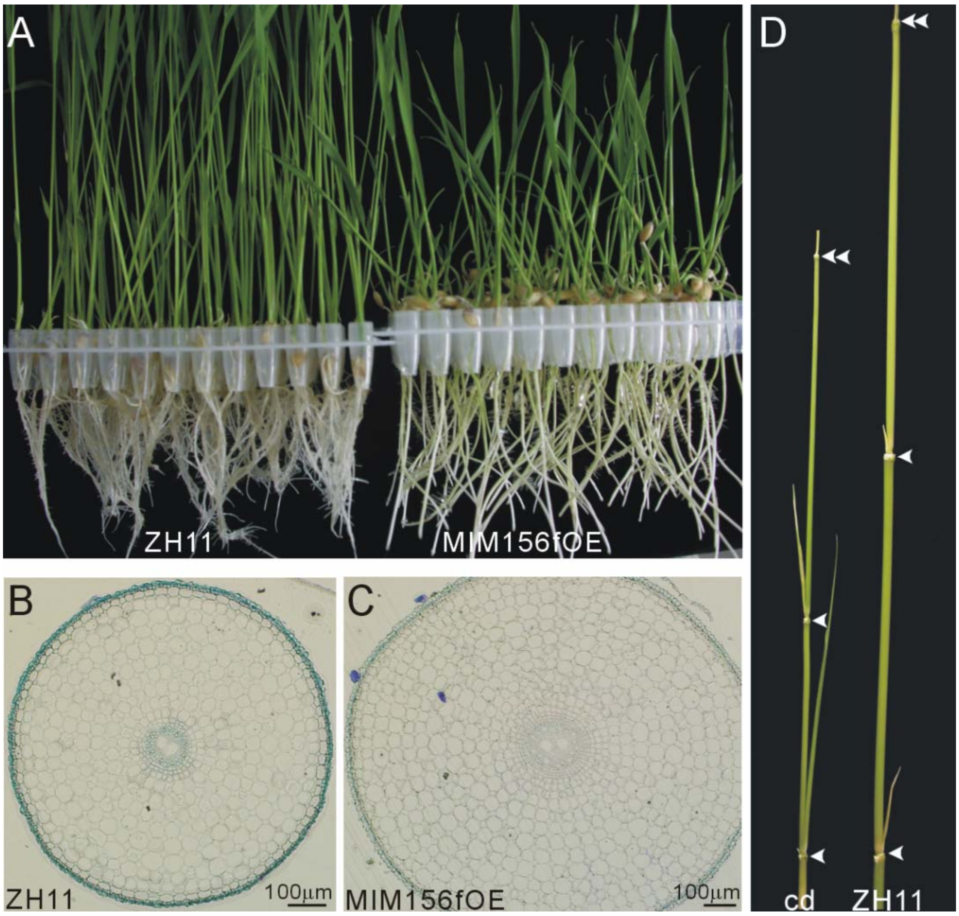

Figure S3

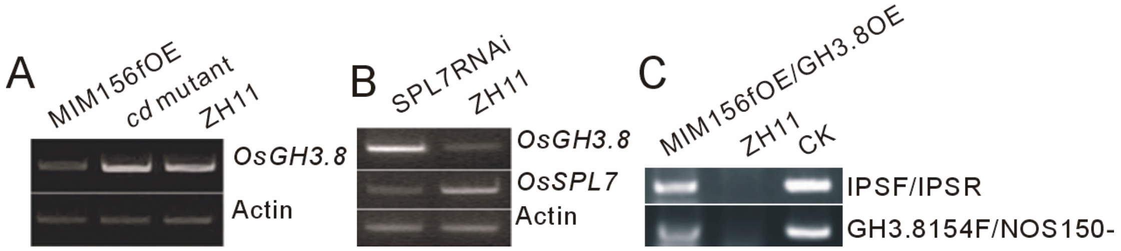
A
MIM156fOE
cd mutant
ZH11
OsGH3.8
Actin
B
SPL7RNAi
ZH11
OsGH3.8
OsSPL7
Actin
C
MIM156fOE/GH3.8OE
ZH11
CK
IPSF/IPSR
GH3.8154F/NOS150-

Figure S4

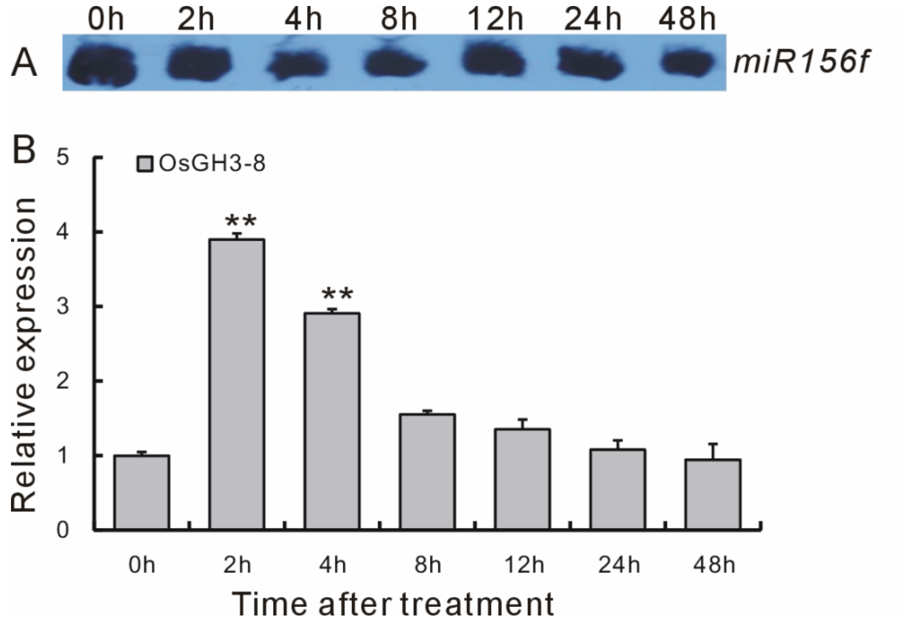
0h
2h
4h
8h
12h
24h
48h
A
miR156f
B
OsGH3-8
Relative expression
5
4
3
2
1
0
**
**
0h
2h
4h
8h
12h
24h
48h
Time after treatment

Figure S5

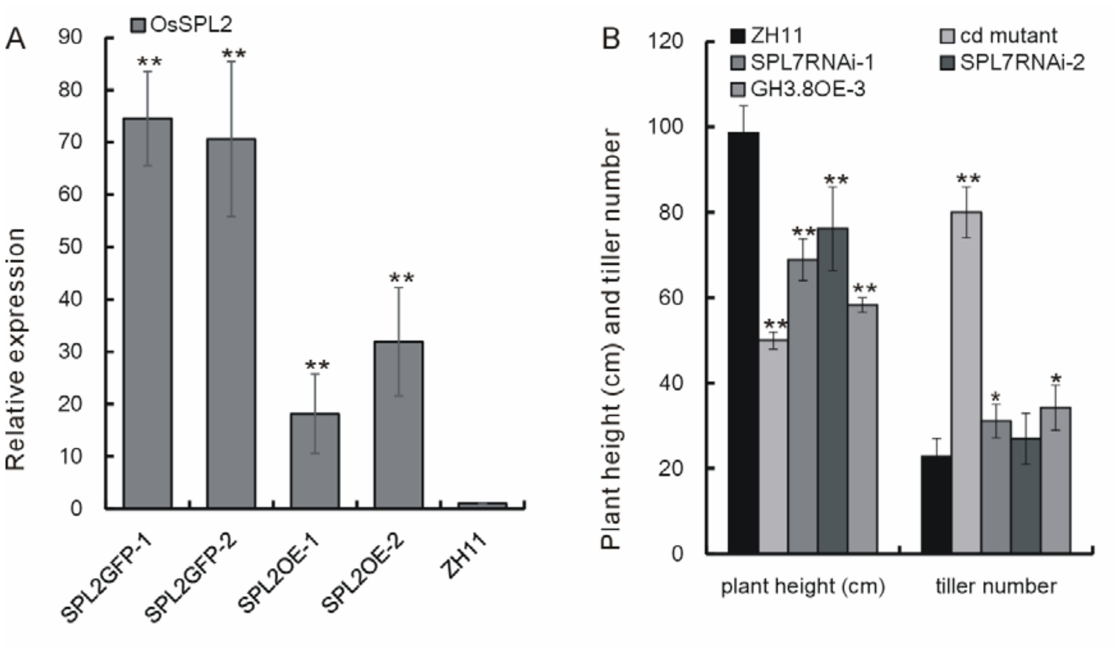
A
OsSPL2
Relative expression
0
10
20
30
40
50
60
70
80
90
**
SPL2GFP-1
SPL2GFP-2
SPL2OE-1
SPL2OE-2
ZH11
B
ZH11
cd mutant
SPL7RNAi-1
SPL7RNAi-2
GH3.8OE-3
Plant height (cm) and tiller number
0
20
40
60
80
100
120
*
plant height (cm)
tiller number
