# Supplementary material for: Modulation of plant architecture by the miR156f–OsSPL7–OsGH3.8 pathway in rice
Source: J Exp Bot. 2018 Jul 25;69(21):5117–30. doi: 10.1093/jxb/ery273 (PMC6184515; doi:10.1093/jxb/ery273)
Supplement: Supplementary Table S2 [file ery273_suppl_supplementary_table_s2.pdf]

Table S2 Analysis of the co-segregation of T-DNA and the phenotype in F2 population of the reciprocal and back cross between WT and the *cd* mutant, experiments were carried out in Summer of Shanghai.

cross	Number of plants	Sensitive to hygromycin	Resistant to hygromycin	
		High(>100cm)	Middle(80-90cm)	Dwarf(<70cm）
cd/ZH11	691	170	352	169
ZH11/cd	792	211	392	189
